# A randomized controlled trial comparing the effectiveness of individual versus household treatment for Scabies in Lambaréné, Gabon

**DOI:** 10.1371/journal.pntd.0008423

**Published:** 2020-06-26

**Authors:** Julian Matthewman, Rella Zoleko Manego, Lia Betty Dimessa Mbadinga, Hana Šinkovec, Katrin Völker, Malik Akinosho, Christian Haedrich, Jeanne Tardif d’Hamonville, Bertrand Lell, Ayola Akim Adegnika, Michael Ramharter, Ghyslain Mombo-Ngoma

**Affiliations:** 1 Centre de Recherches Médicales de Lambaréné (CERMEL), Lambaréné, Gabon; 2 Institute of Tropical Medicine, University of Tübingen, Tübingen, Germany; 3 Department of Tropical Medicine, Bernhard Nocht Institute for Tropical Medicine, & I Dep. of Medicine, University Medical Center Hamburg-Eppendorf, Hamburg, Germany; 4 Center for Medical Statistics, Informatics and Intelligent Systems, Institute of Clinical Biometrics, Medical University of Vienna, Vienna, Austria; 5 Department of Medicine I, Division of Infectious Diseases and Tropical Medicine, Medical University of Vienna, Vienna, Austria; Hebrew University Hadassah Medical School, ISRAEL

## Abstract

**Background:**

It is unclear whether individual treatment of scabies is similarly effective compared to household treatment. This study compared these two treatment strategies with topical benzyl benzoate for treating scabies in Lambaréné, Gabon.

**Methods:**

Participants presenting with uncomplicated scabies were randomized into either the Individual Treatment group, where only the affected participants received treatment, or the Household Treatment group, where all family members were treated in parallel to the affected participants regardless of signs and symptoms. The primary endpoint was clinical cure after 28 days; the secondary endpoint was the proportion of affected household members per household after 28 days.

**Results:**

After 28 days, from a total of 79 participants assessed, 67% (n = 53) were clinically cured; 59% (20/34) in the Individual Treatment group and 73% (33/45) in the Household Treatment group. Participants in the Household Treatment group had about twice the odds of being cured (odds ratio 1.9, 95% confidence interval: 0.8–4.9; p = 0.17). For the secondary outcome, an effect of similar size was observed.

**Conclusions:**

Our findings show that treating close contacts of persons affected by scabies may be beneficial to patients and contacts, however, the benefit was less pronounced than anticipated and further research is needed to definitively answer this question.

## Author summary

Scabies is a skin disease caused by parasitic mites that burrow into the skin and cause itchiness and rash. Since these mites can be easily transmitted from person to person the disease is highly contagious. Many guidelines on the treatment of scabies recommend that all family members and close contacts of persons suffering from scabies should be treated alongside the affected person, in order to prevent the disease from spreading. However, there is only little evidence to be found in the medical literature on how well this practice works. We conducted a study in Lambaréné, Gabon and surrounding villages, where many cases of scabies occurred at the time, to answer this question. We found that, on average, those patients whose entire family was treated were more likely to be cured after 4 weeks, and that fewer of their family members were affected. However, the difference to patients whose families weren’t treated was smaller than expected, suggesting that larger studies are needed to definitively answer the question.

## Introduction

Scabies, a contagious skin infestation caused by the mite *Sarcoptes scabiei* remains an important health issue in the developing world. Its main symptoms are pruritus and typical skin lesions in the form of burrows. Major complications include pyoderma and renal and heart disease, due to secondary bacterial infections. Furthermore, managing the disease can come at significant cost to health care systems[1,2]. Children are the most affected group in the developing world[3].

There are no exhaustive data on scabies prevalence in Gabon, however one can assume that it is endemic as in other tropical regions[1]. The hot and humid climate, often poor sanitation and family members living in crowded living conditions are all factors that favor high scabies transmission.

Several treatments have been demonstrated efficacious for scabies [4–6]. Ivermectin seems to be most effective for large populations with high prevalence of scabies[4]. However the use of ivermectin can lead to serious adverse events in patients with high concentration of *Loa loa* microfilariae in blood[7]. Since *L*. *loa* is endemic in Gabon[8], treatment with ivermectin for scabies should only be used if high microfilarial load can be excluded. Furthermore, only limited safety data exists for ivermectin use for children under 5 years of age or weighing less than 15 kg, a group in which scabies is common[9]. Permethrin is the first line topical treatment for scabies, however it comes at a higher cost per treatment, and is more difficult to obtain in Gabon than benzyl benzoate, a substance which has been previously found to offer good efficacy and safety[4–6]. Benzyl benzoate offers ovicidal activity[10] and has shown good effectiveness as a single dose treatment[11].

It is recommended that the contact persons of an affected individual shall also be treated at the same time, to ensure long term success of treatment, prevent reinfestation and infestation of contact persons[4,9,12]. However, this recommendation lacks a solid background of evidence. An observational study carried out in remote aboriginal communities of northern Australia managed to show a benefit of Household Treatment however this study focused on the initially scabies free family contacts and not the affected participants[12]. A recent review concluded that the effects of treating contact persons to prevent new infestation are unknown[13].

Since 2017, there was anecdotal evidence of an outbreak of itching lesions suggestive of scabies in central Gabon that was named “gratti gratta” by the local population. We conducted a randomized controlled clinical trial using the most commonly used treatment in Gabon, benzyl benzoate, with the aim of evaluating the practice of treating the entire household as compared to treatment of individuals only.

## Methods

A randomized controlled trial was conducted comparing treatment of scabies infected persons only (Individual Treatment Group) to treatment of the entire household (Household Treatment Group), with both groups using the same treatment regimen with benzyl benzoate (NCT04205669). The aim was to assess the practice of systematically recommending Household Treatment and whether it provides a benefit for affected persons by preventing reinfestation.

The number of household members affected 4 weeks after treatment was also assessed, by examining all household members that were present on the day of the visit. Since the presence of itching and skin lesions in a household with a diagnosed scabies case is known to be highly specific [12], we also assessed the number of probable scabies cases per household after treatment according to the judgment of the household responsible person (also referred to as caregiver). In addition, the clinical characteristics of scabies and demographic factors associated with its appearance in Lambaréné, Gabon, and surroundings were assessed.

The trial was designed as a parallel randomized controlled trial with an allocation ratio of 1:1. The trial was conducted according to protocol and no changes to the methods were made after trial commencement.

### Participants

Patients were eligible that were diagnosed with active, uncomplicated scabies and had no known hypersensitivity or allergy against benzyl benzoate. Although previous treatment for scabies was not an exclusion criterion, none of the participants had undergone treatment with an approved scabicide during the preceding 4 weeks. Furthermore, we did not include patients with severe superinfection as this was also considered a contraindication for immediate treatment with topical benzyl benzoate. All participants or their caregivers provided with a signed informed consent form.

The diagnosis of scabies was made by clinical examination by an experienced physician, a method of diagnosis which has been used in a number of other clinical trials to diagnose scabies and has been shown to be highly sensitive and specific. Scabies was defined as the presence of itching together with typical distribution of lesions, such as on the hands, wrists, elbows, axillae, knees, buttocks, genitalia in men, breast areolae in women, palms and soles in children under the age of two[14,15]. A severity scale was used classifying the cases into mild (<10 lesions), moderate (10–50 lesions) and severe (> 50 lesions) according to the number of lesions counted. In case of lesions of unclear origin images were assessed together with a case description via a telemedical consultation by a dermatologist at the Department of Tropical Medicine, Bernhard Nocht Institute for Tropical Medicine, Hamburg, Germany and a recommendation on treatment and on whether to include the patient was given.

Recruitment took place at the Centre de Recherches Médicales de Lambaréné (CERMEL). Cases were identified from patients presenting at the medical research centre, which included patients from screening activities conducted for malaria trials and patients presenting independently due to scabies. The first participant included of a household was defined as the index case. All household members of an index case’s household that presented at the medical research centre (alongside or after the index case) and were also affected were also included as participants.

### Interventions

Index cases were randomized in either of the two treatment groups and participants of the household followed the same treatment as of the index case. In the first group only participants affected by scabies were treated. In the second group all persons living in the same household as the index case were treated, and treatment was dispensed according to the number of household members stated by the caregiver, without any further examination of these non-enrolled household members.

For both groups the choice of treatment was a single application of benzyl benzoate 25% (Ovelle Ltd.), which was mixed with an equal quantity of water for children of up to 12 years of age and with 3 parts of water for infants (<1 year of age) as according to the manufacturer’s instructions. Participants were instructed on the use of the treatment and given a standardized leaflet with instructions, however, the use of the treatment was not supervised and household behavior was not controlled, simulating a real world setting. Participants not cured on the day 28 follow-up visit were given another course of treatment, however they were no longer followed up.

### Outcomes

The primary outcome was clinical cure after 4 weeks with cure being defined as clinical improvement in lesions with no new lesions occurring since treatment application. Photos taken of skin showing characteristic lesions at baseline were used to aid in decision-making and to check for new lesions.

Secondary endpoints included the proportion of scabies cases per household after 4 weeks, any adverse events experienced, distribution of lesions and the association of demographic profiles of participants with the primary outcome.

### Sample size

Since no previous studies were found comparing individual to household treatment the sample size calculation was based on estimates. Estimating a cure rate of 70% and 95% for individual vs. family treatment, respectively, we calculated a sample size of 35 affected participants per group would provide a power of 80% with a two-sided significance level of 0.05. To account for participants being lost to follow-up we aimed to include a total of 100 participants.

### Randomization

Randomization was done in Research Electronic Data Capture (REDCap) software version 8.3.1 (Vanderbilt University, Nashville, TN)[16,17]. An independent statistician created a random allocation table with 80 possible allocations using blocked randomization with random block sizes and provided the file to the REDCap administrator, who uploaded the file, thus, keeping the randomization sequence concealed to the investigators. Eligible participants were enrolled and randomized by the investigators in REDCap creating a non-editable and trackable assignment to one of the two groups. No blinding in regard to interventions was possible due to the nature of the study.

### Statistical methods

Baseline continuous covariates were described by median and interquartile range (IQR) and categorical data by absolute numbers and percentage. The primary analysis was carried out for participants that had completed follow-up (per protocol population) and a secondary efficacy analysis was carried out where we assumed that those who were lost to follow-up were not cured (intention to treat population). Odds ratios (OR) and 95% confidence intervals (95%CI) were calculated to compare the success of household treatment to individual treatment by fitting generalized estimating equations logistic regression that accounts for the correlation between the members of the same family. The marginal treatment effect was estimated from the model with treatment group alone. Additionally, we estimated the stratum specific odds ratios where severity stages and age groups, respectively, were included into the model as stratification variables. To assess the influence of other baseline covariates on the outcome we fitted a multivariable model including severity, age, sex, address and Malaria rapid diagnostic test (RDT) status.

Generalized estimating equations logistic regression was used to compare the per-household odds of being affected by scabies at day 28 between the treatment groups where each household constituted a particular cluster. Two models were fitted, the first including household members according to information given by the caregiver and the second including those household members examined by the investigator. Pearson’s correlation was computed to assess the relationship between the proportion of diseased according to caregiver and the proportion of diseased based on the examination. Two-sided p-values less than 5% were considered statistically significant. Statistical analysis was performed using R 3.4.0 (Vienna, Austria) with packages Zelig (Choirat C, Honaker J, Imai K, King G, Lau O: Zelig: Everyone’s Statistical Software. R package version 5.1.6.1, 2018) and aod (Lesnoff M, Lancelot R: aod: Analysis of Overdispersed Data. R package version 1.3.1, 2012). The outcome assessors were not blinded to treatment assignment.

### Ethics

All adult participants provided written informed consent, and a parent or guardian of any child participant provided written informed consent on the child’s behalf. The study was conducted according to the ethical principles stated in the Declaration of Helsinki, the applicable guidelines for ICH-GCP, and the applicable laws and regulations of Gabon. Approval from the CERMEL ethics review committee was acquired (approval number: CEI-016/2018). The study was conducted in compliance with the study protocol.

### Registration

The trial was registered in the clinicaltrials.gov registry under the Identifier *NCT04205669*. Registration took place retrospectively as the authors were unsure whether the trial fell under the definition of a trial requiring registration. All future trials done by the authors shall be registered prospectively.

## Results

**Fig 1:**
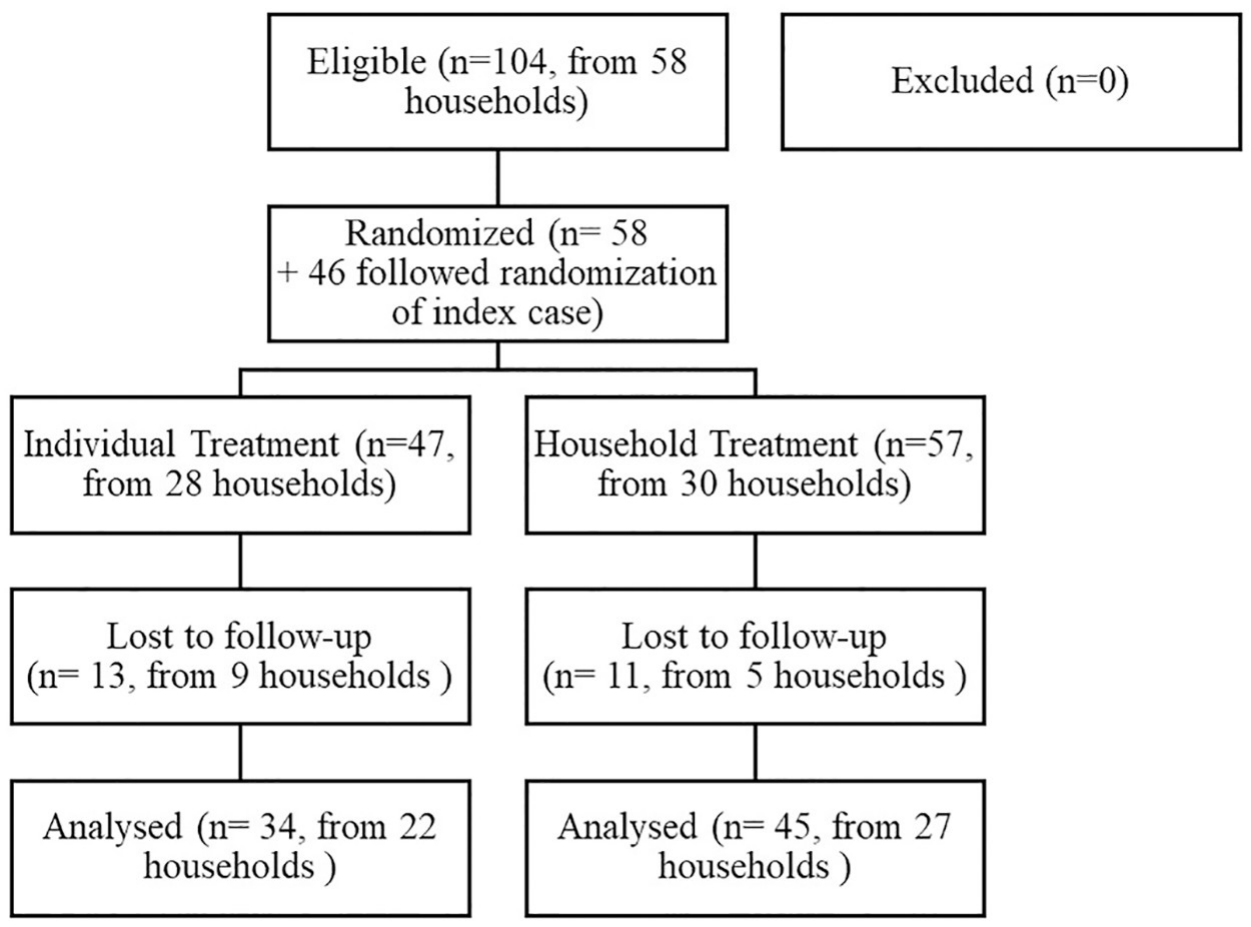
Participant flow diagram.

In total, 104 participants were enrolled and randomized either into the Individual Treatment group (n = 47) or the Household Treatment group (n = 57). The participant flow diagram is shown in Fig 1. For participants where a visit on Day 28 was not possible an attempt to perform the follow-up visit was made within the three following days. If the follow-up visit was not performed until after the third day participants were considered lost to follow-up. Reasons for lost to follow-up were participants moving away, not being reachable by telephone or children being at school at the time of visit. Participants were enrolled from October 2018 to January 2019 and follow-up continued into February 2019. The trial was stopped as soon as the initially calculated sample size had been reached. All participants for whom it was possible to perform the follow-up visit adhered to the randomly assigned treatment. It was not possible to obtain this information for those participants that were lost to follow-up.

Table 1 shows demographic and clinical characteristics of participants. The median age was 9 years ranging from a minimum of 4 months to a maximum of 59 years. 65 participants (63%) were under the age of 12 years. Mild form of scabies was less common than moderate and more severe forms.

**Table 1:** Characteristics of (all 104) participants at baseline.

Characteristic	Individual Treatment (n = 47)	Household Treatment (n = 57)	Total
**Age–No. (%)**			
** • < 12 years**	29 (62%)	36 (63%)	65 (63%)
** • > 12 years**	18 (38%)	21 (37%)	39 (37%)
**Sex–No. (%)**			
** • Male**	22 (47%)	30 (53%)	52 (50%)
** • Female**	25 (53%)	27 (47%)	52 (50%)
**Address–No. (%)**			
** • Rural**	20 (43%)	9 (16%)	29 (33%)
** • Urban**	27 (57%)	48 (84%)	75 (67%)
**Severity–No. (%)**			
** • Mild**	6 (13%)	5 (9%)	11 (11%)
** • Moderate**	19 (40%)	31 (54%)	50 (48%)
** • Severe**	22 (47%)	21 (37%)	43 (41%)
**Malaria RDT–No. (%)**			
** • Positive**	22 (47%)	16 (28%)	38 (37%)
** • Negative**	25 (53%)	41 (72%)	76 (63%)

Of the 58 index cases, 30 (52%) presented with one or more, and an additional 12 (21%) with two or more affected household members who were then also included as participants. Household size was evaluated on the Day 28 follow-up visit for the available 47 out of 58 families. The median household size was 7 (IQR 6–8) in the Individual Treatment group and 7 (IQR 6–10) in the Household Treatment group. The maximum household size was 22.

Out of the 79 participants assessed on the Day 28 follow-up visit, 67% (n = 53) of participants were cured; 59% (n = 20) out of 34 participants in the Individual Treatment group and 73% (n = 33) out of the 45 participants in the Household Treatment group (OR: 1.9, 95% CI: 0.8–4.9; p = 0.17). The corresponding estimated risk difference was 0.14 (95% CI: -0.06–0.35; p = 0.17). Table 2 shows the effect estimates stratified by severity and age.

**Table 2:** Number of participants in each stratum (n), Odds Ratios (OR) with 95% confidence intervals (CI) for marginal treatment effect and stratified by severity and age group.

	n	OR	95% CI	p-value
**Household treatment**	79	1.9	0.8–4.9	0.17
**Stratified by severity**				
• **Mild**	9	2	0.1–35.8	0.64
• **Moderate**	36	4.2	0.9–19.9	0.07
• **Severe**	34	1	0.2–4.5	1
**Stratified by age**				
• <**12**	51	1.6	0.5–5.6	0.44
• >**12**	28	2.7	0.5–15.7	0.28

In an additional analysis including all participants (n = 104) and assuming that participants lost to follow-up were not cured, 51% (n = 53) of participants were cured; 43% (20/47) in the Individual Treatment group and 58% (33/57) in the Household Treatment group (OR: 1.9, 95% CI: 0.8–4.4; p = 0.16). The corresponding estimated risk difference was 0.15 (95% CI: -0.04–0.34; p = 0.17).

Table 3 shows the results of a multivariable analysis, to assess the influence of other baseline covariates (age, sex, location, severity, number of household members, positive malaria RDT) on the outcome. The adjusted OR of being cured at Day 28 were 2.3 for the Household Treatment group as compared to the Individual Treatment group. Having a case of scabies defined as moderate or severe or being over the age of 12 were significantly associated with achieving a cure. Since the number of household members was assessed on the Day 28 follow-up visit, this variable is not included in the multivariable analysis due to scarce data.

**Table 3:** Results of multivariable generalized estimating equations logistic regression analysis. Odds Ratios (OR) (of achieving a cure) and confidence intervals (CI).

	OR	95% CI	p-value
**Individual Treatment** **Household Treatment**	1 2.3	0.8–6.5	0.12
**Age < 12** **Age ≥ 12**	1 4.8	1.7–13.6	0.02
**Male sex** **Female sex**	1 1.2	0.4–3.3	0.81
**Rural address** **Urban address**	1 1.1	0.4–3.0	0.94
**Mild severity** **Moderate severity**	1 9.2	3.2–26.4	0.05
**Severe severity**	20.3	7.1–58.0	0.02
**Negative Malaria RDT** **Positive Malaria RDT**	1 1.9	0.7–5.4	0.39

The median prevalence of scabies per household on Day 28 was 33% (in total 40 out of 100) in the Individual Treatment group and 27% (in total 34 out of 136) in the Household Treatment group (OR: 0.5, 95% CI: 0.2–1.2; p = 0.11), calculated based on cases identified by clinical diagnosis by the investigator. Calculated based on the judgment of the caregiver the median prevalence was 22% (in total 47 out of 153) and 21% (in total 56 out of 211) respectively (OR: 0.8, 95% CI: 0.4–1.8; p = 0.61).

**Fig 2:**
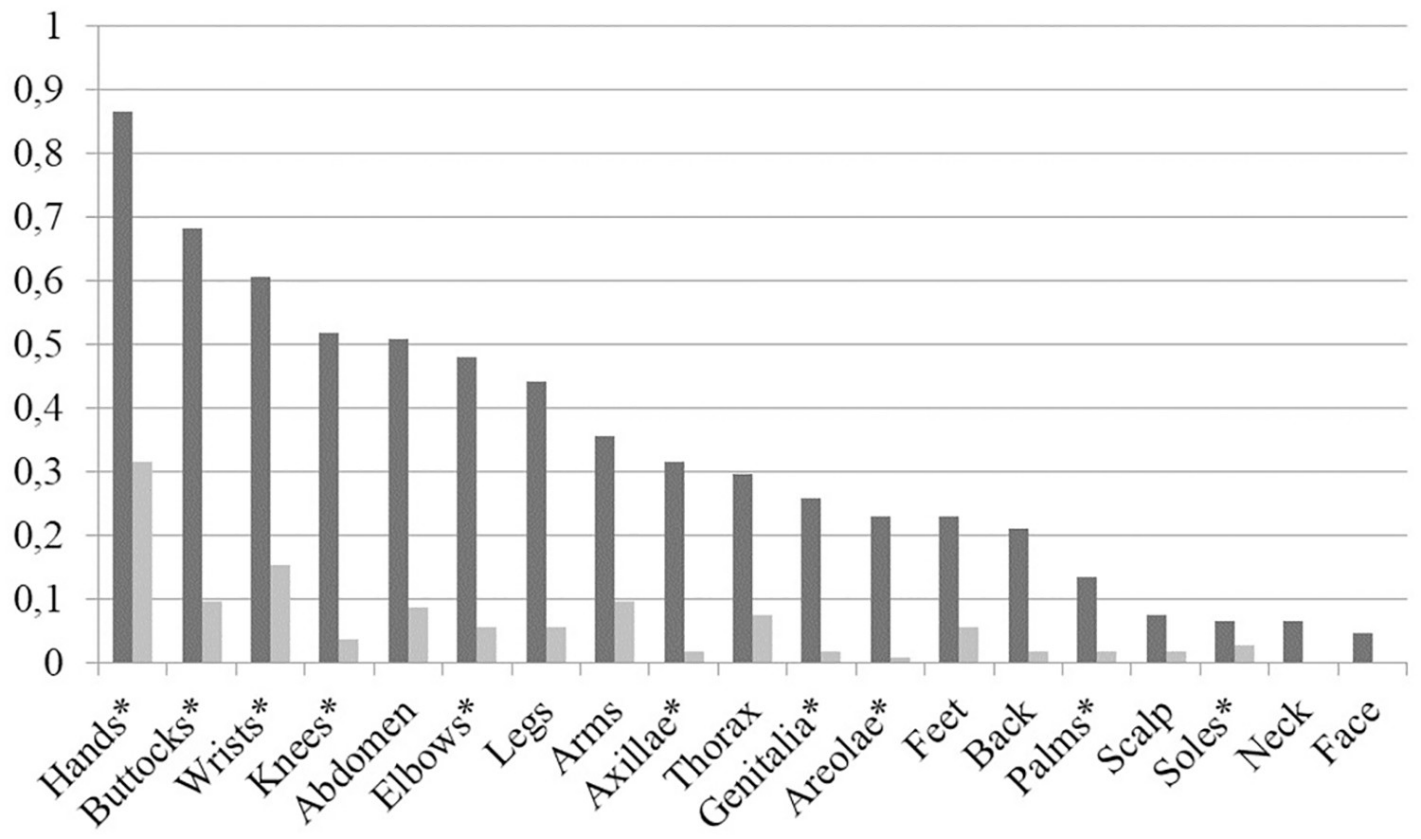
Lesion Distribution by frequency. The first(darker) bar shows D0 and the second(lighter) bar shows D28. “Hands” and “Feet” include lesions in the interdigital and interphalangeal folds and exclude lesions on Palms and Soles respectively. Lesions which were considered as typical for scabies are marked with *.

Fig 2 shows the distribution of lesions assessed at inclusion and on the Day 28 follow-up visit. Hands and buttocks were the sites most commonly affected on Day 0 whereas hands and wrists were the sites most commonly affected on Day 28. Five of the six participants with mild severity of scabies at baseline that were not considered cured at Day 28 showed lesions at locations not affected at baseline.

The first (darker) bar shows D0 and the second (lighter) bar shows D28. “Hands” and “Feet” include lesions in the interdigital and interphalangeal folds and exclude lesions on Palms and Soles respectively. Lesions which were considered as typical for scabies are marked with *

## Discussion

This study primarily aimed at assessing the benefit of Household Treatment for patients and their family contacts in a real world setting. To adequately assess this, an effectiveness study was designed simulating most closely real world settings. The odds of achieving a cure in the Household Treatment group were almost twice those of the Individual Treatment group(OR: 1.9, 95% CI: 0.8–4.9), indicating that Household Treatment could be associated with treatment success. The proportion of household members affected after 28 days was also lower in the Household Treatment group. However, due to too small sample size and lower than expected clearing rates, these differences did not reach statistical significance.

In the analysis stratified by severity and age, there was some evidence that household treatment is associated with a cure in cases with moderate severity. However, these results should be interpreted cautiously due to the sparse data.

Multivariable analysis was performed as secondary effectiveness outcome indicating higher cure rates in patients with moderate and severe scabies. This finding may be explained by higher compliance, with participants being more likely to apply the topical treatment to the entire body, and not just affected sites, if more sites were affected. Among the 6 participants with mild severity of scabies at baseline that were not considered cured at Day 28, five did in fact show new lesions at locations not affected at baseline. Duration of disease might also play a role in achieving cure, which ostensibly would have been shorter in patients with mild scabies. The possibility of misdiagnosis must also be taken into account, which could also have been more likely in mild scabies. No other studies were found showing a significant effect of severity at baseline on treatment success.

Another characteristic associated with achieving cure was being in the group of participants aged 12 years or older. Hygiene and interaction with other children are possible explanations. Although according to the manufacturer’s instructions, it is also possible that the diluted treatment (1:1 for < 12 years of age or 3:1 for <1 year of age) given to the younger age groups played a role in decreased treatment success. One should note that young children generally constitute the group most affected by scabies in the developing world[1].

The overall cure rate of 67% found in this study lies somewhat below the rate found in some efficacy studies for benzyl benzoate and other established treatments such as ivermectin[4,6]. A possible reason for treatment failure is the application of benzyl benzoate only once. Although this single dose treatment regimen was according to manufacturer’s instructions and has been used in other studies[4], a regimen with multiple applications might have been more effective. The compliance of participants was not enforced or assessed, as to replicate a real world setting, which might however provide a further explanation for higher rates of treatment failure than those seen in some efficacy studies. Possible factors benefiting reinfestation in our setting are high prevalence, large families and high mobility within communities. Importantly, there are several determinants of unsupervised effectiveness of treatments with efficacy being only one of them.

In our study population family size is large with some households having as many as 22 members living in the same house. Furthermore, there is significant mobility in communities with individuals often changing household. It was not assessed if the composition of the household at Day 28 was the same as at the baseline visit. The prevalence of scabies per household based on numbers given by the caregiver was lower than the prevalence observed by the investigator, indicating that Scabies is an underestimated or underreported problem in our setting.

Our findings on lesion distribution were similar to findings from other trials[18]. It has to be taken into account that scabies typical lesions were a diagnostic criterion, thus predetermining the lesion distribution to a certain extent.

Due to this being the first study on this research question, a limitation of the study was that effectiveness of interventions had to be estimated for sample size calculation based on previous efficacy studies on scabies treatment and an assumed difference according to treatment strategy. With the actual effectiveness lower than anticipated and the observed difference between treatment groups being smaller than estimated this study lacks statistical power in the primary outcome. However, we believe that the effect observed in the primary outcome and the secondary outcome analysis indicate a benefit of household treatment. Further research, taking into account the smaller difference of outcomes in this setting, is warranted.

Affected household contacts of participants belonging to the Individual Treatment group that presented at the medical research centre by themselves or alongside the index case had to also be treated and enrolled, which further diminished the difference between the two treatment groups.

Due to time and resource constraints only clinical features and not dermatoscopy or skin scrapings were used for the diagnosis of scabies and definition of cure, which may be considered as a further limitation of this study. Clinical diagnosis is however well established for scabies and has been used in several previous studies[4]. To minimize assessor differences all participants were seen by the same physician. If there was any doubt about whether lesions seen were actually typical for scabies, electronic pictures of lesions were sent to a dermatologist at the Bernhard Nocht Institute for Tropical Medicine for confirmation. Pictures of lesions were also used to aid with definition of cure. A potential source of bias was that the outcome assessors were not blinded to treatment assignment.

76 participants presented with at least one other affected family member and an additional 7 participants with typical lesions on male genitalia thus 83 cases or 79.8% can be considered as “clinical scabies” according to consensus criteria established by the International Alliance for the Control of Scabies (IACS)[19]. One can assume that the actual proportion of “clinical scabies” would have been higher when assessing if any other family members were affected at baseline, but this was not done due to the study design.

## Conclusion

In this randomized controlled clinical trial participants who were instructed and given sufficient topical therapy to treat their household contacts were more likely to be cured after 28 days and a lower proportion of individuals was affected after 28 days in those participants’ families. However, the benefits were modest and overall effectiveness was lower than expected. This highlights the difficulties of treating scabies in a setting such as that of our study. Being the first study of its kind, more research in this field is needed. Our findings are supportive of current recommendations on treatment of family contacts of scabies affected persons.
